# The effect of piglet vitality, birth order, and blood lactate on the piglet growth performances and preweaning survival

**DOI:** 10.1186/s40813-022-00299-2

**Published:** 2022-12-23

**Authors:** Md Karim Uddin, Shah Hasan, Olli Peltoniemi, Claudio Oliviero

**Affiliations:** Department of Production Animal Medicine, Faculty of Veterinary Medicine, University of Helsinki, Helsinki, Finland

**Keywords:** Piglets, Vitality, Blood lactate, Colostrum intake, Birth order, Mortality rate

## Abstract

**Background:**

Litter size is going up in modern pig production. Due to large litter size and increased farrowing duration, newborn piglets, during parturition, can suffer from asphyxiation. This alters their blood energy parameters and can contribute to their reduced vitality at birth, which is detrimental to their growth performance and survival. We aimed to evaluate the effects of vitality score, piglets’ umbilical cord blood lactate, glucose, and butyrate, and birth order on growth performance and the preweaning mortality of piglets.

**Results:**

The more vital (vitality score 2, VS2) piglets had higher umbilical cord blood glucose and butyrate, lower blood lactate, and higher colostrum intake (CI) at birth, and showed higher body weight at birth and at weaning than did less vital piglets (vitality score 1, VS1). Umbilical cord lactate negatively correlated with vitality, colostrum intake, and growth before weaning. Among the four birth-order groups (BOGs), piglets born earlier during parturition had a higher mean vitality score than those born later. BOG1 and BOG2 had significantly higher CI (*p* < 0.05) than BOG3 and BOG4.

**Conclusion:**

Changes in piglets’ body weight, colostrum intake, and umbilical cord lactate are associated with piglet vitality and asphyxiation during farrowing. In addition, asphyxiation induced higher umbilical cord lactate may serve as an indicator of low vitality and low colostrum consumption. Overall, improvement in piglets’ vitality and in the farrowing process can help in reducing piglet mortality.

## Introduction

In modern pig production, increasing litter size is associated with reduced uterine blood flow in sows [1]. As a result of decreased blood flow, fetuses received reduced amounts of nutrients, resulting in an increase in the presence of low-birth-weight piglets with low energy reserves [2, 3]. When piglets are born deficient in energy reserves, right after birth they have higher nutritional requirements for maintenance of their thermoregulation and physical activity [4]. In addition, their limited ability to cope with environmental stressors such as cold, disease, and limited nutrition availability predisposes such piglets to relatively high rates of neonatal morbidity and mortality [5]. In modern pig production farms, insufficient colostrum intake has been identified as one of the major causes of neonatal mortality and decreased weight gain, especially in low birthweight piglets [6–8]. Fetal hypoxia during the process of farrowing is another problem, considered to be the reason for reduced piglet vitality in the first few hours of life and for an increased stillbirth rate [9]. Some sow- and piglet factors contributing to the risk of hypoxia are long farrowing duration, large litter size, excessive uterine contractions of sows, low birthweight piglets, and being born later in the piglet’ birth order [9, 10]. It has been suggested that 15–20% of liveborn piglets could also be born after suffering from hypoxia, with minimal suckling activity after birth, which lowers their ingestion of sufficient colostrum for homeostasis and survival [11]. Moreover, increased farrowing duration may be associated with repeated uterine contractions and compression of the placental blood supply, resulting in reduced fetal oxygenation [11, 12]. This condition can even be worsened by farmers trying to speed up delayed farrowing by injecting the sow with repeated or too-high dosages of oxytocin [13, 14]. In fetuses during parturition, reduced oxygenation causes a drop in heart rate and initiates anaerobic metabolism, which collectively reduces blood pH and increases blood lactate, originating from cardiac and skeletal muscle [11, 15]. Piglet blood lactate is regarded as an indicator of fetal hypoxia, and it is also inversely related to the ability of the piglets to thermoregulate during an acute cold stress [12, 16, 17]. An increased level of umbilical cord lactate is positively correlated with lower colostrum consumption [18]. BHB is thought to be one of the end products of accelerated hepatic mitochondrial oxidation of fatty acids, and its plasma level is regarded as an indication of ketogenesis [19]. It is a mechanism whereby ketone bodies are produced mainly in the mitochondrial matrix of liver cells and are transported to other organs, i.e., the brain, to meet the energy demands [20, 21]. Research has revealed that an increased level of BHB may improve cerebral energy status and neuroprotection during hypoxia ischemia (HI) in the rat [22]. HI is regarded as a brain injury that occurred due to asphyxia during birth, and might cause developmental delay, cognitive impairment, and cerebral palsy in infants [23, 24]. In newborn piglets, studies have shown that BHB levels remain low [25]. Due to hyper-prolific sows producing large litters, piglet birth order becomes another important factor to consider. Large litters are associated with increased numbers of low-birth-weight piglets, and longer farrowing durations [26–28]. The longer farrowing leads to an increased risk of asphyxiation in those piglets expelled late in the birth order, because undergoing longer and stronger uterine contractions may cause early rupture of the umbilical cord [29, 30]. In addition, piglets born late in the birth order are forced to consume a lower amount of colostrum, and its immunoglobulins than early birth piglets do [28, 31].

Piglet vitality refers to the strength and vigor of piglets, both necessary for their survival [14, 32, 33]. Researchers have used various methods to assess piglet vitality [32–34]. Most are complex, time-consuming, and often not feasible for field-study settings. Piglet vitality can be measured on a vitality scale (0–3, increasing vitality with an increasing score), by considering the behavior of piglets within 15 s of their birth [32]. Studies exploring the effects of piglet vitality on piglet performance and survival are, however, scarce. We hypothesize that production performance and pre-weaning survival of piglets are associated with their vitality at birth, birth order, and blood-parameter level. We therefore aimed to evaluate the effects of piglet vitality, birth order, and piglet blood parameter levels’ on piglet performance and survival until weaning.

## Results

### Effect of piglet vitality

**Fig. 1:**
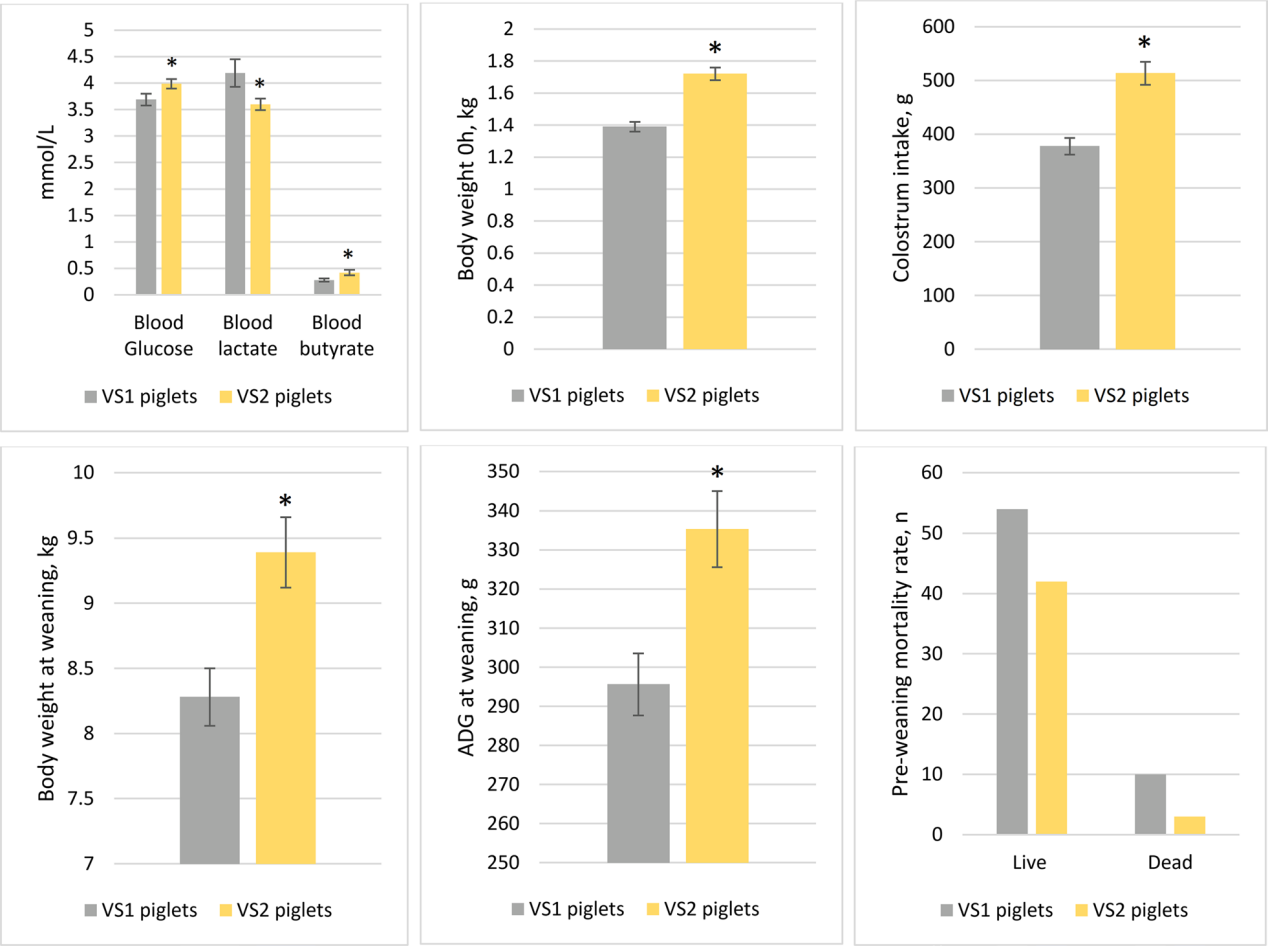
Effects of piglet’ vitality on blood parameters, and piglet performance before weaning. VS1, low vitality piglets with score 1; VS2, higher vitality piglets with score 2; BW, body weight; BW0h, body weight at birth; ADG, average daily gain. The results are presented as means and SEM. For pre-weaning mortality rate values are expressed as number of cases. Asterisks indicate the statistically significant differences (*p* < 0.05)

No piglets presented with vitality score 0 (VS0) and vitality score 3 (VS3). We observed a significant difference between the VS1 and VS2 piglets (*p* < 0.01). VS2 piglets had significantly higher body weight at birth (BW0h), and higher umbilical cord blood glucose and butyrate than did VS1 piglets (*p* < 0.01), but umbilical cord blood lactate level was higher in VS1 than in VS2 piglets. In addition, by 24 h, VS2 piglets consumed significantly higher amounts of colostrum than did VS1 piglets (*p* < 0.01). At weaning, VS2 piglets were of a higher body weight (*p* < 0.01) and had a higher ADG than did VS1 piglets (Fig. 1). We observed a numerical difference in preweaning mortality, being higher for VS1 piglets (n = 10) (*p* > 0.05) than VS2 piglets (n = 3) (Fig. 1).

### Effects of piglet birth order

The average total duration of farrowing (from the first piglet to the last piglet) was 354.60 ± 191.89 min (mean ± standard deviation). The average duration of farrowing for the different birth order categories (BOGs) is described in Table 1. The mean vitality score was the highest in BOG-2 (1.44 ± 0.07) and the lowest in BOG-4 (1.14 ± 0.14, *p* < 0.05). Umbilical cord lactate level was higher in BOG-4 (6.28 ± 1.37 mmol/L), and lower in BOG-3 (3.59 ± 0.18 mmol/L). However, glucose level tended to be higher in BOG-4 (4.07 ± 0.35 mmol/L) than in BOG-3 (3.59 ± 0.16 mmol/L). BOG-1 piglets consumed the largest amount of colostrum (455.72 ± 23.94 g), and BOG-4 the lowest (338.95 ± 37.12 g, *p* < 0.05) (Table 1). No differences appeared in mean butyrate level, body weight to weaning, ADG to weaning, and pre-weaning mortality rate among birth order groups (Table 1).

**Table 1:** Association of birth orders with piglet traits

Characteristics	BOG-1 (n = 50)	BOG-2 (n = 48)	BOG-3 (n = 35)	BOG-4 (n = 7)	*p* value
Farrowing duration, min	354.6 ± 26.00^a^	355.13 ± 27.09^a^	368.77 ± 36.50^a^	456 ± 107^a^	0.63
Birth interval, min	29.66 ± 7.32^a^	17.72 ± 3.69^a^	14.23 ± 2.84^a^	73.43 ± 30.62^b^	0.002
Mean vitality score	1.42 ± 0.07^a^	1.44 ± .07 ^b^	1.26 ± 0.07^a^	1.14 ± 0.14^a^	**0.06**
BW0h, kg	1.53 ± 0.04^a^	1.51 ± 0.05^a^	1.50 ± 0.04^a^	1.46 ± 0.13 ^a^	0.65
Umbilical cord blood glucose, mmol/L	4.01 ± 0.12^a^	3.75 ± 0.12^a^	3.59 ± 0.16^a^	4.07 ± 0.35^a^	0.10
Umbilical cord blood lactate, mmol/L	4.01 ± 0.31^a^	3.76 ± 0.13^a^	3.59 ± 0.18^a^	6.28 ± 1.37^b^	**0.01**
Umbilical cord blood butyrate, mmol/L	0.32 ± 0.05^a^	0.39 ± .05^a^	0.34 ± .06^a^	0.25 ± 0.15^a^	0.91
CI, g	455.72 ± 23.94^b^	437.86 ± 21.0^a^	392.73 ± 29.48^a^	338.95 ± 37.12^a^	**0.05**
BW weaning, kg	9.25 ± 0.35^a^	8.38 ± 0.280^a^	8.65 ± .32^a^	9.07 ± 0.56^a^	0.23
ADG weaning, g	330.31 ± 12.61^a^	299.26 ± 10.03^a^	308.90 ± 11.33^a^	324.17 ± 20.14^a^	0.23
Pre-weaning mortality, n	4^a^	4^a^	4^a^	1^a^	0.98

The results are presented as means ± standard error mean (SEM)

BOG-1, birth order group-1; BOG-2, birth order group-2; BOG-3, birth order group-3; BOG-4, birth order group-4; BW, body weight; BW0h, body weight at birth; CI, colostrum intake

Different letters indicate the statistically significant differences between groups (*p* < 0.05)

### Effect of piglet umbilical cord blood lactate

**Fig. 2:**
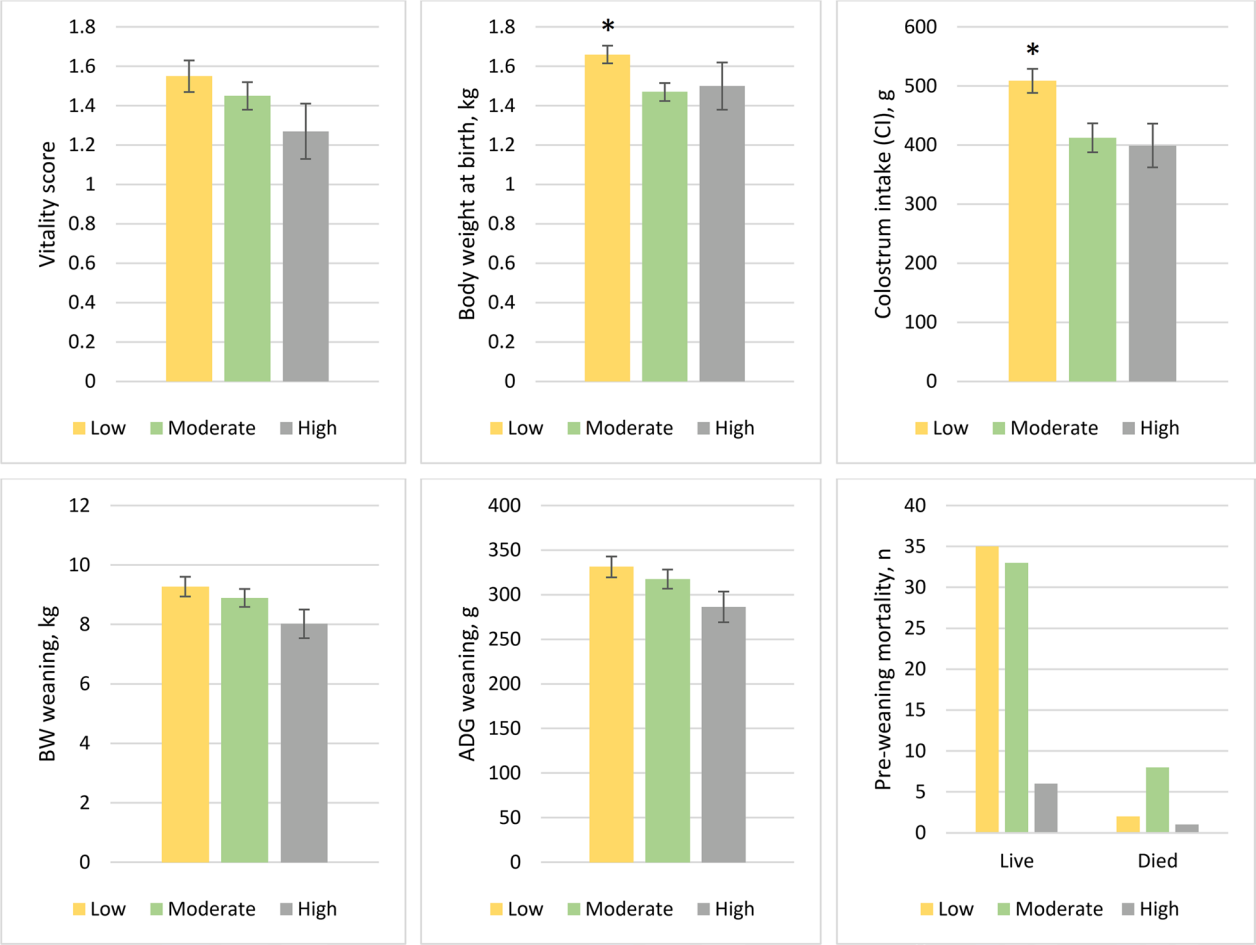
Effects of umbilical cord blood lactate on vitality scores, colostrum intake, body weight, and preweaning mortality rate. Body weight of piglets at birth (BW0h) and colostrum intake (CI) is higher in low lactate group. The results are presented as means ± standard error of mean. Low = < 3.5 mmol/L lactate, moderate = 3.5–5.0 mmol/L lactate, high = > 5.0 mmol/L lactate; BW, body weight; BW0h, body weight at birth; CI, colostrum intake. Asterisks indicate the statistically significant differences (*p* < 0.05)

Regarding piglet umbilical cord lactate levels, mean vitality score tended to be raised in piglets in the low-lactate group (1.55 ± 0.07) more than in high-lactate groups (1.27 ± 0.14; *p* = 0.11). Higher BW0h was in the low-lactate group than in the high- and moderate-lactate groups (*p* < 0.05). However, low-lactate piglets consumed the maximum amount of colostrum (508.70 ± 20.42 g; *p* < 0.05) compared to that of moderate- and high-lactate piglets. At weaning, body weight and ADG tended to be higher in low-lactate piglets than in high-lactate piglets (*p* < 0.1). Moreover, the latter had numerically higher mortality than the former piglets (Fig. 2). In a multivariate linear model of piglets' weaning body weight with vitality scores and blood lactate levels, we observed that one unit increase in blood lactate level tended to decrease 0.42 kg of weaning body weight for piglets. In contrast, one unit increase in vitality scores increased the piglets' body weight to weaning by 0.71 kg (Table 2).

**Table 2:** Multivariate mixed effect linear model of weaning body weight, umbilical cord lactate, and vitality score

Outcome variable	Predictor variables	Coefficient	Standard error	95% conf. interval	*p* value
Body weight at weaning, kg	Lactate	− 0.43	0.27	− 0.95 to 0.10	0.11
	Vitality score	0.71	0.38	− 0.03 to 1.4	0.06

## Discussion

We found that our higher-vitality piglets had higher colostrum intake, higher body weight at birth and at weaning, and higher weaning ADG than did low-vitality piglets. Early-born piglets had high vitality and consumed a higher amount of colostrum than did those later born. Moreover, piglets with high umbilical cord blood-lactate levels had a lower birthweight, showed less vitality, and consumed a lower amount of colostrum than did those with low umbilical cord blood lactate.

Piglet vitality refers to the physical strength of newborn piglets [14, 32, 33]. Among factors associated with piglet vitality [33, 35], and performance an important one is birth weight [36]. Birthweights of our VS1 piglets were lower than those of VS2 piglets. Due to their low energy reserve [37] and low vitality [12], low-birthweight piglets tend to have their first suckle of colostrum later than that of their heavier littermates [5, 36, 38]. Delay in attachment to the sow udder for suckling causes, in low-birthweight piglets, insufficient colostrum consumption [39]. In our study, low-vitality piglets (VS1) consumed about 135 g less colostrum than did those with high vitality (VS2). This difference may mean that the more vigorous VS2 piglets could more easily reach the udder and produce better pressure during suckling, allowing them to consume more colostrum than the less vigorous VS1 piglets. Inadequate colostrum intake results in inadequate passive immunity and malnourishment, followed by poor growth performance [36, 40]. In addition, low colostrum is inversely correlated with umbilical cord blood lactate [41]. Piglets with low blood glucose at birth are at greater risk of mortality 7 days after birth [36]. Perhaps lower glycogen level in newborn piglets may be associated with low viability [36]. Umbilical cord blood glucose was lower in VS1 than in VS2 piglets. Likely because VS2 piglets had a higher energy reserve.

At different stages of production, piglets’ birthweight is positively correlated with body growth [36]. Higher birthweight results in faster body growth at weaning [40, 42, 43]. In our study, birth weights of VS2 piglets were higher than of VS1 piglets, and at weaning, their body weight and ADG were also higher than those of VS1 piglets. This may be due to high-vitality piglets consuming a greater amount of colostrum, providing more growth metabolites and immunomodulators, which facilitate their intestinal development and consequently bodily growth [28, 44]. Beta-hydroxy butyrate in plasma is regarded as an indication of ketogenesis [20], which is found at a low level in newborn piglets [25]. Surprisingly, higher-vitality piglets had higher umbilical cord blood butyrate levels than did low vital piglets, suggesting that ketogenesis is prominent in high vital piglets. This butyrate elevation may be a factor in providing neuroprotection by improving cerebral energy status during hypoxia ischemia (HI) [22] to the high-vitality piglets, which is important for healthy brain functioning in their later life.

Blood lactate is derived from glucose in the absence of oxygen [45]. Consequently, when piglets are suffering from hypoxia during birth, umbilical cord plasma lactate increases [12]. Langendijk et al. [46] assessed lactate level as an indirect asphyxia indicator at birth. In accordance with our present findings, their piglets that experienced asphyxia during parturition showed decreased colostrum intake. In addition, Trujillo-Ortega et al. [47] reported blood-lactate levels higher in those piglets surviving severe-to-moderate intrapartum asphyxia than in piglets born with mild or no evidence of intrapartum asphyxia; the latter is more likely due to secondary hypoxia than to changes in glucose metabolism. We observed that elevated umbilical cord blood-lactate levels significantly reduced individual piglets’ colostrum intake, which supports earlier findings that being born with increased lactate levels can predispose piglets to less colostrum ingestion [41]. In our study, for piglets born later in the farrowing process (BOG 4), this might be attributed to the increased farrowing duration leading to repeated uterine contractions and compression of the placenta and the uterus together with their blood vessels [11, 12]. This condition can reduce fetal oxygen supply, causing a drop in heart rate and more production of lactate via anaerobic metabolism [11, 15]. These hypoxic piglets may need more time to adapt to the extrauterine environment and to achieve contact with the udder than do normal piglets. This delay in udder contact and the competition with their littermates results in less colostrum intake. Surprisingly, an inverse correlation exists in piglets between low birth weight and degree of asphyxia [12]. This means the lower the birth weight, the higher the lactate level. Our findings support the notion that piglets with higher levels of umbilical cord blood lactate have lower birth weights than do piglets with lower levels. This may be due to the fact that small piglets are more likely to experience perinatal hypoxia due to placental insufficiency [12, 48]. In addition, piglet vitality is inversely related to this asphyxiation [17, 34]. Accordingly, in our study, the vitality score was higher in those piglets with low levels of umbilical cord lactate than in those with high levels. We also observed piglets with higher umbilical cord blood lactate as having numerically higher death cases than did those with lower lactate. This supports findings that lactate levels have been higher in piglets who died before 3 weeks of age [12, 49].

Inclusion of hyper-prolific sows through genetic selection improves litter size and concurrently lengthens farrowing duration [50, 51]. Studies have demonstrated that piglets born later in the farrowing process experience asphyxia, which reduces their vitality and consumption of colostrum [12]. Our data concurs with these results: piglets of the first birth order group are more vital and consume more colostrum than do later-born piglets. These may be due to the piglets’ taking a shorter time to reach the udder, and with increased vitality at birth, and fighting less with littermates, and thus achieving higher colostrum consumption [36, 52].

We found later-born piglets to have higher umbilical cord lactate levels than those early born. This may result from their longer stay in the uterus and birth canal. As a result, they experience prolonged asphyxia, increased hypoxia, decreased heart rate, and produce high lactate [11, 15].

All these findings imply that the increased farrowing duration of hyper-prolific sows leads to the risk during parturition, of piglet asphyxiation. This prolonged asphyxiation raises lactate production, which lowers piglet vitality in the extrauterine environment. Being unable to consume adequate colostrum, these piglets of less vitality have reduced transfer of immunoglobulin and growth factors from their mother, which affects production performance and survival. This is supported by our findings in which piglets with low umbilical cord blood lactate levels at birth had the best ADG and weight at weaning.

Earlier findings by Stefaficzyk-Krzymowska et al. [53] indicate that fetuses at the tip of a uterine horn could be in a more favorable position for development and growth, due to their closer proximity to the ovary and therefore their stronger progesterone milieu compared with that of fellow fetuses at a more basal position in the uterine horn. Consequently, those piglets born late in the birth order, such as the last birth order group in our study, should be heavier than those piglets born first. Based on our data, however, this was apparently not the case, since no difference emerged between BOG1 and BOG4. Therefore, at least in the data from our moderately prolific sows, it seems that relative position in the uterus and relative proximity of the fetus to the ovary seems not to significantly influence fetal development and growth. On the other hand, it appears clear that those piglets originating from the tip of a uterine horn are those that are subject to repeated prolonged contractions of the uterus and thereby most severe asphyxia during the process of parturition, therefore explaining the apparent lack of viability of the last piglets born in the birth order [11]. Other factors, such as placental texture, aggressiveness of the sow, interval between fetuses and placental areolae suggest that the success of the process is more depending on the dam than the fetus [54]. However, there should be more in-depth studies involving the labor-intensive process of labeling placentae at birth to explore the risk factors of low viability of piglets at birth. We have a few limitations in our study. One limitation is that we conducted this study with a limited number of sows. Another limitation is that we did not measure some piglet characteristics, like meconium scoring and umbilical cord characteristics, for vitality assessment. However, meconium staining of the piglets was observed very seldom in this study. In addition, we did not have stillbirth, and farrowing duration was in the range of 5–7 h.

## Conclusions

Our data supports the hypothesis that changes in body weight, colostrum intake, and umbilical cord lactate may be due to changes in piglet vitality, and probably in increased asphyxia during parturition (especially for those piglets born in the last birth order group, BOG-4). Moreover, we also found higher umbilical cord lactate as an indicator of low vitality and low colostrum consumption. Furthermore, we found a positive association between early-born piglets and piglet vitality, blood lactate, and colostrum intake. The genetic line we used was less prolific than that of other hyperprolific lines in Finland and Europe. One would therefore expect even more of the effects seen here in hyperprolific sows. Our own findings could contribute to focusing on appropriate interventions to improve outcomes, e.g., via measuring cord blood lactate and assisting less vital piglets, which could improve their growth and survival.

## Materials and methods

The experiment was conducted from April, 2020 to June, 2020, on a commercial pig farm in Kauhava, Finland.

### Sow selection and feeding

On a piglet-producing farm, we included 140 piglets from 10 sows. Sows belonged to the Figen muscle synthetic sire line (Figen Oy, Pietarsaari, Finland) developed from Finnish Landrace and Finnish Large White breeds, and the average parity was 3.1 ± 0.31. We allocated sows in two batches at a 2-week interval. Then we transferred them to farrowing crates about 7 days before the expected farrowing days. Each farrowing crate was equipped with feeders and waterers for sows and piglets. Sows and piglets received liquid and dry feeds. The daily average feed intake during gestation and the lactation period was 2.83 ± 0.05 kg for gestating sows and 6.43 ± 0.21 kg for lactating sows. The feed composition is presented in the table (Table 3). The principal investigator was present himself. In addition, sows were monitored and video recorded starting from 3 days before their expected farrowing days until 24 h after start of farrowing. None of the sows included in the study received a medical induction of farrowing. Average farrowing duration was 354.6 ± 191.89 min (mean ± standard deviation). Obstetrical intervention was applied in one sow, when birth interval exceeded 1 h. If piglets were moved to other sows 24 h after birth, to balance litters, they were excluded from the study. In this study, average total piglets born was 14.0 ± 2.54 piglets per sow with no stillbirth occurrences.

**Table 3:** Composition of sow diet during gestation and lactation

Ingredients	Gestating sow	Lactating sow
Dry matter, %	87.2	87.9
Crude protein, %	11.5	17.5
Crude fat, %	2.5	4.2
Crude fiber, %	5.5	3.7
Ash, %	5.7	6.3
NE, MJ	9.1	10.1
Lysine, g	5.1	8.9
Threonine, g	3.8	6.1
Methionine, g	3.6	5.3
Calcium, g	8.5	10.2
Phosphorus, g	5.1	5.7
Fusible phosphorus, g	3.2	3.8
Sodium, g	2.0	2.6
Selenium, mg	0.5	0.5
Vitamin A, IU	10.7	11.9
Vitamin D3, IU	1.8	2.0
Vitamin E, mg	109.1	127.0

### Piglet sample collection

We visually assessed piglets’ vitality immediately after birth, on a 4-category scale (0–3) described by Baxter et al. [32]. The vitality scores (VS) are 0 = when there is no movement, no breathing after 15 s, stillbirth (VS0, dead piglets); 1 = no movement within 15 s but piglet was breathing or attempting to breathe (VS1 piglet); 2 = When piglet showed little movement within 15 s, breathing or attempting to breathe (VS2 piglets); and 3 = When piglet had good movement, good breathing, and piglet attempted to stand within 15 s (VS3 piglet).

We observed no VS0, and VS3 piglets during vitality score assessment. After vitality score assessment, we collected umbilical cord blood with a 2-ml Henke-Ject® disposable syringe and a 21-G needle (Henke-Sass, Wolf GmbH, Germany) from the intact or slightly broken umbilical cord. In other cases, we collected the required amount of blood (3–4 drops) by pressing the cord backwards with two fingertips for quick strip testing of blood glucose, lactate, and butyrate. Immediately after birth, after drying the back of each piglet with a paper towel, we marked each one on its back with a black marker according to its birth order. After marking the piglets, we measured their body weight with a commercial weighing scale (XL-Float-22, Patriot ®, Finland), and then each piglet was replaced in the position from which was lifted. We tested piglet umbilical-cord blood glucose (CentriVet, Acon Laboratories, USA), lactate (Statstrip Lactate Xpress Meter, Woodley Equipment Company Ltd., UK), and butyrate (CentriVet, Acon Laboratories, USA) using rapid testing kits. The kits were used so carefully that when they came into contact with fluids other than blood, they gave error results. After 24 h from the first piglet’s birth, we individually weighed all piglets again to calculate their colostrum intake, and then ear-tagged them. We monitored and recorded the farrowing activities using Denver IP cameras (SHO-110, Denver Electronics®, Denmark). For litter balancing, piglets were allowed to be moved to other sows only after the colostrum calculation was completed, and only those piglets remaining with their own mother were followed until 4 weeks of age. We categorized the piglets into 4 birth-order groups (BOG) according to their birth order: BOG1 = 1–5, BOG2 = 6–10, BOG3 = 11–15, and BOG4 = 16–20. Other piglet parameters recorded were weight at weaning, average daily gain (ADG) from birth to weaning, and pre-weaning mortality rate (%).

### Colostrum intake

We estimated colostrum yield (CY) by summing individual piglets’ colostrum intake (CI) within a litter, by the following regression formula developed by Theil et al. [55].

$${\text{CI}},\;{\text{g}} = {-}{1}0{6} + {2}.{26}\;{\text{WG}} + {2}00\;{\text{BWb}} + 0.{111}\;{\text{D}}{-}{1},{414}\;{\text{WG}}/{\text{D}} + 0.0{182}$$

WG = weight gain in 24 h; BWb = body weight at birth in kg; and D = duration of colostrum suckling (time ranging from the individual piglet’s birth until 24 h after the 1st piglet was born) expressed in minutes.

### Statistical analysis

Data analysis was done by Stata 17.0 (Stata MP/17 for Windows; Stata Corp., College Station, TX, USA). Data were expressed as mean ± SEM. For the vitality score, we used t-test to observe the difference between vitality scores. To observe the difference between groups of umbilical cord lactate level and birth order, we used ANOVA. After finding significant difference in ANOVA, we used the Tukey test to observe the specific group's means (compared with each other) which were different. We used a chi-square test to observe the differences in mortality between groups of vitality scores, lactate categories, and BOGs. Level of significance we reported at a *p* value of < 0.05 and trend as < 0.1 [56]. We performed a univariate analysis for each variable as a basis for building up a generalized linear (GLM) model. A preliminary full model was created with explanatory variables with *p* ≤ 0.2. To fit a final model, we performed a stepwise backward elimination procedure. We used the “meglm” command in stata to run a GLM model of weaning body weight with lactate level and vitality scores (1 and 2 only, since no piglet showed vitality scores 0 and 3), where sow id, total birth, and trial batches were included as random factors in the model. Piglet parameters were birth interval, pre-weaning mortality, BW0h, body weight at 24 h (BW24), weaning body weight, and average daily gain (ADG) at weaning (28 days). Piglets were divided into three categories: low blood lactate level (< 3.5), moderate blood lactate (3.5–5.0), and high blood lactate (> 5.0), based on their umbilical cord blood lactate at birth.

## Data Availability

Data related to this study can be accessible by request to the corresponding author.
